# Supplementary material for: MicroRNAs in Leukemias: A Clinically Annotated Compendium
Source: Int J Mol Sci. 2022 Mar 23;23(7):3469. doi: 10.3390/ijms23073469 (PMC8998245; doi:10.3390/ijms23073469)

Table S1

Disease type	Gene/miRNA name
Leukemia	FANCD2
Leukemia	ATM
Leukemia	FANCC
Leukemia	FANCE
Leukemia	BRIP1
Leukemia	BLM
Leukemia	NRAS
Leukemia	FANCA
Leukemia	FANCG
Leukemia	FANCF
Acute megakaryocytic leukemia	GATA1
Acute M1 myeloid leukemia	FLT3
Acute M1 myeloid leukemia	NPM1
Acute promyelocytic leukemia	STAT5B
Acute promyelocytic leukemia	IRF2BP2
Acute promyelocytic leukemia	NUMA1
Acute promyelocytic leukemia	ZBTB16
Acute promyelocytic leukemia	RARA
Acute promyelocytic leukemia	PML
Acute promyelocytic leukemia	PRKAR1A
Acute promyelocytic leukemia	NPM1
Childhood Acute Lymphoblastic Leukemia	IKZF1
Childhood Acute Lymphoblastic Leukemia	NBN
Childhood Acute Lymphoblastic Leukemia	CDKN2A
Chronic Lymphocytic Leukemia	TP53
Chronic Lymphocytic Leukemia	LEF1
Chronic Lymphocytic Leukemia	ATM
Chronic Lymphocytic Leukemia	P2RX7
Chronic Lymphocytic Leukemia	IRF4
Chronic Lymphocytic Leukemia	SF3B1
Chronic Lymphocytic Leukemia	PLCG2
Chronic Lymphocytic Leukemia	BCL2
Chronic Lymphocytic Leukemia	IGHV3-21
Chronic Lymphocytic Leukemia	POT1
Chronic Neutrophilic Leukemia	CSF3R
Hairy Cell Leukemia	BRAF
Juvenile Myelomonocytic Leukemia	KRAS
Juvenile Myelomonocytic Leukemia	RRAS
Juvenile Myelomonocytic Leukemia	PTPN11
Juvenile Myelomonocytic Leukemia	NF1
Juvenile Myelomonocytic Leukemia	SETBP1
Juvenile Myelomonocytic Leukemia	ARHGAP26

Juvenile Myelomonocytic Leukemia	NRAS
Juvenile Myelomonocytic Leukemia	CBL
Juvenile Myelomonocytic Leukemia	ASXL1
Mast-Cell Leukemia	KIT
Acute Myelocytic Leukemia	TP53
Acute Myelocytic Leukemia	CBFB
Acute Myelocytic Leukemia	CEBPA
Acute Myelocytic Leukemia	MLF1
Acute Myelocytic Leukemia	HRAS
Acute Myelocytic Leukemia	RUNX1
Acute Myelocytic Leukemia	CREBBP
Acute Myelocytic Leukemia	KIT
Acute Myelocytic Leukemia	MYH11
Acute Myelocytic Leukemia	KMT2A
Acute Myelocytic Leukemia	SETBP1
Acute Myelocytic Leukemia	NUP214
Acute Myelocytic Leukemia	FLT3
Acute Myelocytic Leukemia	FANCC
Acute Myelocytic Leukemia	FANCE
Acute Myelocytic Leukemia	SBDS
Acute Myelocytic Leukemia	CSF3R
Acute Myelocytic Leukemia	BRIP1
Acute Myelocytic Leukemia	NRAS
Acute Myelocytic Leukemia	CBL
Acute Myelocytic Leukemia	FANCA
Acute Myelocytic Leukemia	SPI1
Acute Myelocytic Leukemia	NSD1
Acute Myelocytic Leukemia	WT1
Acute Myelocytic Leukemia	DNMT3A
Acute Myelocytic Leukemia	IDH1
Acute Myelocytic Leukemia	IDH2
Acute Myelocytic Leukemia	NPM1
Acute Myelocytic Leukemia	TERT
Acute Myelocytic Leukemia	FANCG
Acute Myelocytic Leukemia	PALB2
Acute Myelocytic Leukemia	FANCF
Acute Myelocytic Leukemia	MECOM
Acute Myelocytic Leukemia	GATA2
Acute Myelocytic Leukemia	JAK2
Acute Myelocytic Leukemia	KRAS
Acute Myelocytic Leukemia	SF3B1
Acute Myelocytic Leukemia	MLLT10
Acute Myelocytic Leukemia	ETV6
Acute Myelocytic Leukemia	TET2

Acute Myelocytic Leukemia	PTPN11
Acute Myelocytic Leukemia	FANCD2
Acute Myelocytic Leukemia	GFI1
Chronic Atypical BCR-ABL Negative Myeloid Leukemia	STETBP1
Chronic Atypical BCR-ABL Negative Myeloid Leukemia	CSF3R
Chronic Myelomonocytic Leukemia	TET2
Chronic Myelomonocytic Leukemia	KDM6A
Chronic Myeloid Leukemia	JAK2
Chronic Myeloid Leukemia	ABL1
Chronic Myeloid Leukemia	SETBP1
Chronic Myeloid Leukemia	BCR
Precursor Cell Lymphoblastic Leukemia-Lymphoma	PAX5
Precursor Cell Lymphoblastic Leukemia-Lymphoma	ABL1
Precursor Cell Lymphoblastic Leukemia-Lymphoma	SH2B3
Precursor Cell Lymphoblastic Leukemia-Lymphoma	IKZF1
Precursor Cell Lymphoblastic Leukemia-Lymphoma	NBN
Precursor Cell Lymphoblastic Leukemia-Lymphoma	CRLF2
Precursor Cell Lymphoblastic Leukemia-Lymphoma	FLT3
Precursor Cell Lymphoblastic Leukemia-Lymphoma	BCR
Precursor T-Cell Lymphoblastic Leukemia-Lymphoma	ABL1
Precursor T-Cell Lymphoblastic Leukemia-Lymphoma	FBXW7
Precursor T-Cell Lymphoblastic Leukemia-Lymphoma	LMO1
Precursor T-Cell Lymphoblastic Leukemia-Lymphoma	RPL5
Precursor T-Cell Lymphoblastic Leukemia-Lymphoma	TAL1
Precursor T-Cell Lymphoblastic Leukemia-Lymphoma	RPL10
Precursor T-Cell Lymphoblastic Leukemia-Lymphoma	CNOT3
Precursor T-Cell Lymphoblastic Leukemia-Lymphoma	MYB
Precursor T-Cell Lymphoblastic Leukemia-Lymphoma	TLX1
Precursor T-Cell Lymphoblastic Leukemia-Lymphoma	PHF6
Precursor T-Cell Lymphoblastic Leukemia-Lymphoma	IL7R
Precursor T-Cell Lymphoblastic Leukemia-Lymphoma	NOTCH1
Leukemia	hsa-mir-125a
Leukemia	hsa-mir-125b-1
Leukemia	hsa-mir-125b-2

Leukemia	hsa-mir-138-1
Leukemia	hsa-mir-138-2
Leukemia	hsa-mir-143
Leukemia	hsa-mir-150
Leukemia	hsa-mir-15a
Leukemia	hsa-mir-16-1
Leukemia	hsa-mir-16-2
Leukemia	hsa-mir-17
Leukemia	hsa-mir-181a-1
Leukemia	hsa-mir-181a-2
Leukemia	hsa-mir-18a
Leukemia	hsa-mir-196b
Leukemia	hsa-mir-199a-1
Leukemia	hsa-mir-199a-2
Leukemia	hsa-mir-199b
Leukemia	hsa-mir-19a
Leukemia	hsa-mir-19b-1
Leukemia	hsa-mir-19b-2
Leukemia	hsa-mir-20a
Leukemia	hsa-mir-21
Leukemia	hsa-mir-27a
Leukemia	hsa-mir-31
Leukemia	hsa-mir-331
Leukemia	hsa-mir-339
Leukemia	hsa-mir-34a
Leukemia	hsa-mir-378a
Leukemia	hsa-mir-454
Leukemia	hsa-mir-485
Leukemia	hsa-mir-495
Leukemia	hsa-mir-575
Leukemia	hsa-mir-660
Leukemia	hsa-mir-663a
Leukemia	hsa-mir-9-1
Leukemia	hsa-mir-9-2
Leukemia	hsa-mir-9-3
Leukemia	hsa-mir-92a-1
Leukemia	hsa-mir-92a-2
Acute leukemia	hsa-mir-222
B-cell leukemia	hsa-mir-125b-1
B-cell leukemia	hsa-mir-125b-2
B-cell leukemia	hsa-mir-142
B-cell leukemia	hsa-mir-155
B-cell leukemia	hsa-mir-15a
B-cell leukemia	hsa-mir-16-1

B-cell leukemia	hsa-mir-17
B-cell leukemia	hsa-mir-18a
B-cell leukemia	hsa-mir-19a
B-cell leukemia	hsa-mir-19b-1
B-cell leukemia	hsa-mir-20a
B-cell leukemia	hsa-mir-92a-1
Acute biphenotypic leukemia	hsa-mir-125b-1
Acute biphenotypic leukemia	hsa-mir-182
Acute biphenotypic leukemia	hsa-mir-584
Acute lymphoblastic leukemia	hsa-mir-221
Chronic lymphoblastic leukemia	hsa-mir-34a
Chronic lymphocytic B-cell leukemia	hsa-mir-103a-1
Chronic lymphocytic B-cell leukemia	hsa-mir-103a-2
Chronic lymphocytic B-cell leukemia	hsa-mir-106b
Chronic lymphocytic B-cell leukemia	hsa-mir-107
Chronic lymphocytic B-cell leukemia	hsa-mir-125b-1
Chronic lymphocytic B-cell leukemia	hsa-mir-125b-2
Chronic lymphocytic B-cell leukemia	hsa-mir-130a
Chronic lymphocytic B-cell leukemia	hsa-mir-146a
Chronic lymphocytic B-cell leukemia	hsa-mir-146b
Chronic lymphocytic B-cell leukemia	hsa-mir-148a
Chronic lymphocytic B-cell leukemia	hsa-mir-151a
Chronic lymphocytic B-cell leukemia	hsa-mir-155
Chronic lymphocytic B-cell leukemia	hsa-mir-15a
Chronic lymphocytic B-cell leukemia	hsa-mir-15b
Chronic lymphocytic B-cell leukemia	hsa-mir-16-1
Chronic lymphocytic B-cell leukemia	hsa-mir-16-2
Chronic lymphocytic B-cell leukemia	hsa-mir-17
Chronic lymphocytic B-cell leukemia	hsa-mir-181a-1
Chronic lymphocytic B-cell leukemia	hsa-mir-181a-2
Chronic lymphocytic B-cell leukemia	hsa-mir-181b-1
Chronic lymphocytic B-cell leukemia	hsa-mir-181b-2
Chronic lymphocytic B-cell leukemia	hsa-mir-181c
Chronic lymphocytic B-cell leukemia	hsa-mir-187
Chronic lymphocytic B-cell leukemia	hsa-mir-18a
Chronic lymphocytic B-cell leukemia	hsa-mir-195
Chronic lymphocytic B-cell leukemia	hsa-mir-196b
Chronic lymphocytic B-cell leukemia	hsa-mir-19a
Chronic lymphocytic B-cell leukemia	hsa-mir-19b-1
Chronic lymphocytic B-cell leukemia	hsa-mir-206
Chronic lymphocytic B-cell leukemia	hsa-mir-20a
Chronic lymphocytic B-cell leukemia	hsa-mir-21
Chronic lymphocytic B-cell leukemia	hsa-mir-22
Chronic lymphocytic B-cell leukemia	hsa-mir-221

Chronic lymphocytic B-cell leukemia	hsa-mir-222
Chronic lymphocytic B-cell leukemia	hsa-mir-223
Chronic lymphocytic B-cell leukemia	hsa-mir-23b
Chronic lymphocytic B-cell leukemia	hsa-mir-24-1
Chronic lymphocytic B-cell leukemia	hsa-mir-27b
Chronic lymphocytic B-cell leukemia	hsa-mir-29a
Chronic lymphocytic B-cell leukemia	hsa-mir-29b-1
Chronic lymphocytic B-cell leukemia	hsa-mir-29b-2
Chronic lymphocytic B-cell leukemia	hsa-mir-29c
Chronic lymphocytic B-cell leukemia	hsa-mir-342
Chronic lymphocytic B-cell leukemia	hsa-mir-34a
Chronic lymphocytic B-cell leukemia	hsa-mir-34b
Chronic lymphocytic B-cell leukemia	hsa-mir-34c
Chronic lymphocytic B-cell leukemia	hsa-mir-640
Chronic lymphocytic B-cell leukemia	hsa-mir-650
Chronic lymphocytic B-cell leukemia	hsa-mir-92a-1
Chronic myelogenous Bcr-abl positive leukemia	hsa-mir-144
Chronic myelogenous Bcr-abl positive leukemia	hsa-mir-155
Chronic myelogenous Bcr-abl positive leukemia	hsa-mir-181a-1
Chronic myelogenous Bcr-abl positive leukemia	hsa-mir-212
Chronic myelogenous Bcr-abl positive leukemia	hsa-mir-30a
Chronic myelogenous Bcr-abl positive leukemia	hsa-mir-31
Chronic myelogenous Bcr-abl positive leukemia	hsa-mir-328
Chronic myelogenous Bcr-abl positive leukemia	hsa-mir-369
Chronic myelogenous Bcr-abl positive leukemia	hsa-mir-410
Chronic myelogenous Bcr-abl positive leukemia	hsa-mir-451a
Chronic myelogenous Bcr-abl positive leukemia	hsa-mir-451b
Chronic myelogenous Bcr-abl positive leukemia	hsa-mir-564
Chronic myelogenous Bcr-abl positive leukemia	hsa-mir-615
Chronic myelogenous Bcr-abl positive leukemia	hsa-mir-663a
Myeloid leukemia	hsa-mir-125b-1
Myeloid leukemia	hsa-mir-125b-2
Myeloid leukemia	hsa-mir-130a
Myeloid leukemia	hsa-mir-130b
Myeloid leukemia	hsa-mir-138-1
Myeloid leukemia	hsa-mir-138-2
Myeloid leukemia	hsa-mir-17
Myeloid leukemia	hsa-mir-18a

Myeloid leukemia	hsa-mir-19a
Myeloid leukemia	hsa-mir-19b-1
Myeloid leukemia	hsa-mir-19b-2
Myeloid leukemia	hsa-mir-20a
Myeloid leukemia	hsa-mir-23a
Myeloid leukemia	hsa-mir-32
Myeloid leukemia	hsa-mir-92a-1
Myeloid leukemia	hsa-mir-92a-2
Acute myeloid leukemia	hsa-let-7a-1
Acute myeloid leukemia	hsa-let-7a-2
Acute myeloid leukemia	hsa-let-7a-3
Acute myeloid leukemia	hsa-let-7b
Acute myeloid leukemia	hsa-let-7c
Acute myeloid leukemia	hsa-let-7d
Acute myeloid leukemia	hsa-let-7e
Acute myeloid leukemia	hsa-let-7f-1
Acute myeloid leukemia	hsa-let-7f-2
Acute myeloid leukemia	hsa-let-7g
Acute myeloid leukemia	hsa-let-7i
Acute myeloid leukemia	hsa-mir-100
Acute myeloid leukemia	hsa-mir-10a
Acute myeloid leukemia	hsa-mir-125b-1
Acute myeloid leukemia	hsa-mir-126
Acute myeloid leukemia	hsa-mir-127
Acute myeloid leukemia	hsa-mir-142
Acute myeloid leukemia	hsa-mir-143
Acute myeloid leukemia	hsa-mir-145
Acute myeloid leukemia	hsa-mir-146a
Acute myeloid leukemia	hsa-mir-150
Acute myeloid leukemia	hsa-mir-154
Acute myeloid leukemia	hsa-mir-155
Acute myeloid leukemia	hsa-mir-15a
Acute myeloid leukemia	hsa-mir-15b
Acute myeloid leukemia	hsa-mir-17
Acute myeloid leukemia	hsa-mir-181a-1
Acute myeloid leukemia	hsa-mir-181a-2
Acute myeloid leukemia	hsa-mir-181b-1
Acute myeloid leukemia	hsa-mir-181b-2
Acute myeloid leukemia	hsa-mir-181c
Acute myeloid leukemia	hsa-mir-181d
Acute myeloid leukemia	hsa-mir-18a
Acute myeloid leukemia	hsa-mir-193a
Acute myeloid leukemia	hsa-mir-193b
Acute myeloid leukemia	hsa-mir-196a-1

Acute myeloid leukemia	hsa-mir-196a-2
Acute myeloid leukemia	hsa-mir-196b
Acute myeloid leukemia	hsa-mir-199b
Acute myeloid leukemia	hsa-mir-19a
Acute myeloid leukemia	hsa-mir-19b-1
Acute myeloid leukemia	hsa-mir-19b-2
Acute myeloid leukemia	hsa-mir-203
Acute myeloid leukemia	hsa-mir-20a
Acute myeloid leukemia	hsa-mir-219-1
Acute myeloid leukemia	hsa-mir-219-2
Acute myeloid leukemia	hsa-mir-221
Acute myeloid leukemia	hsa-mir-222
Acute myeloid leukemia	hsa-mir-223
Acute myeloid leukemia	hsa-mir-224
Acute myeloid leukemia	hsa-mir-24-1
Acute myeloid leukemia	hsa-mir-25
Acute myeloid leukemia	hsa-mir-26a-1
Acute myeloid leukemia	hsa-mir-26a-2
Acute myeloid leukemia	hsa-mir-27a
Acute myeloid leukemia	hsa-mir-299
Acute myeloid leukemia	hsa-mir-29a
Acute myeloid leukemia	hsa-mir-29b-1
Acute myeloid leukemia	hsa-mir-29b-2
Acute myeloid leukemia	hsa-mir-30c-1
Acute myeloid leukemia	hsa-mir-30c-2
Acute myeloid leukemia	hsa-mir-3151
Acute myeloid leukemia	hsa-mir-323a
Acute myeloid leukemia	hsa-mir-337
Acute myeloid leukemia	hsa-mir-342
Acute myeloid leukemia	hsa-mir-34a
Acute myeloid leukemia	hsa-mir-34b
Acute myeloid leukemia	hsa-mir-370
Acute myeloid leukemia	hsa-mir-382
Acute myeloid leukemia	hsa-mir-424
Acute myeloid leukemia	hsa-mir-590
Acute myeloid leukemia	hsa-mir-628
Acute myeloid leukemia	hsa-mir-663a
Acute myeloid leukemia	hsa-mir-92a-1
Acute myeloid leukemia	hsa-mir-92a-2
Acute myeloid leukemia	hsa-mir-98
Chronic-phase myeloid leukemia	hsa-mir-10a
Chronic-phase myeloid leukemia	hsa-mir-29a
Chronic-phase myeloid leukemia	hsa-mir-29b-1
Chronic-phase myeloid leukemia	hsa-mir-29b-2

Chronic-phase myeloid leukemia	hsa-mir-30a
Acute promyelocytic leukemia	hsa-mir-125b-1
Acute promyelocytic leukemia	hsa-mir-181a-1
Acute promyelocytic leukemia	hsa-mir-181a-2
Acute promyelocytic leukemia	hsa-mir-181b-1
Acute promyelocytic leukemia	hsa-mir-181b-2
Acute promyelocytic leukemia	hsa-mir-181d
Acute promyelocytic leukemia	hsa-mir-223
Acute promyelocytic leukemia	hsa-mir-34a
Acute promyelocytic leukemia	hsa-mir-34b
Acute promyelocytic leukemia	hsa-mir-34c
Adult T-cell leukemia/lymphoma	hsa-mir-142
Adult T-cell leukemia/lymphoma	hsa-mir-155
Adult T-cell leukemia/lymphoma	hsa-mir-16-1
Adult T-cell leukemia/lymphoma	hsa-mir-16-2
Adult T-cell leukemia/lymphoma	hsa-mir-17
Adult T-cell leukemia/lymphoma	hsa-mir-18a
Adult T-cell leukemia/lymphoma	hsa-mir-19a
Adult T-cell leukemia/lymphoma	hsa-mir-19b-1
Adult T-cell leukemia/lymphoma	hsa-mir-19b-2
Adult T-cell leukemia/lymphoma	hsa-mir-203
Adult T-cell leukemia/lymphoma	hsa-mir-205
Adult T-cell leukemia/lymphoma	hsa-mir-20a
Adult T-cell leukemia/lymphoma	hsa-mir-326
Adult T-cell leukemia/lymphoma	hsa-mir-451a
Adult T-cell leukemia/lymphoma	hsa-mir-663a
Adult T-cell leukemia/lymphoma	hsa-mir-711
Adult T-cell leukemia/lymphoma	hsa-mir-92a-1
Adult T-cell leukemia/lymphoma	hsa-mir-92a-2
Precursor B-cell lymphoblastic leukemia/lymphoma	hsa-mir-125b-1
Precursor B-cell lymphoblastic leukemia/lymphoma	hsa-mir-126
Precursor B-cell lymphoblastic leukemia/lymphoma	hsa-mir-142
Precursor B-cell lymphoblastic leukemia/lymphoma	hsa-mir-222
Precursor B-cell lymphoblastic leukemia/lymphoma	hsa-mir-339
Precursor B-cell lymphoblastic leukemia/lymphoma	hsa-mir-373
Precursor B-cell lymphoblastic leukemia/lymphoma	hsa-mir-451a
Precursor cell lymphoblastic leukemia/lymphoma	hsa-let-7b
Precursor cell lymphoblastic leukemia/lymphoma	hsa-mir-100
Precursor cell lymphoblastic leukemia/lymphoma	hsa-mir-125b-1

Precursor cell lymphoblastic leukemia/lymphoma	hsa-mir-125b-2
Precursor cell lymphoblastic leukemia/lymphoma	hsa-mir-126
Precursor cell lymphoblastic leukemia/lymphoma	hsa-mir-128-2
Precursor cell lymphoblastic leukemia/lymphoma	hsa-mir-143
Precursor cell lymphoblastic leukemia/lymphoma	hsa-mir-146a
Precursor cell lymphoblastic leukemia/lymphoma	hsa-mir-17
Precursor cell lymphoblastic leukemia/lymphoma	hsa-mir-181a-1
Precursor cell lymphoblastic leukemia/lymphoma	hsa-mir-181a-2
Precursor cell lymphoblastic leukemia/lymphoma	hsa-mir-181b-1
Precursor cell lymphoblastic leukemia/lymphoma	hsa-mir-181c
Precursor cell lymphoblastic leukemia/lymphoma	hsa-mir-182
Precursor cell lymphoblastic leukemia/lymphoma	hsa-mir-196b
Precursor cell lymphoblastic leukemia/lymphoma	hsa-mir-221
Precursor cell lymphoblastic leukemia/lymphoma	hsa-mir-223
Precursor cell lymphoblastic leukemia/lymphoma	hsa-mir-29a
Precursor cell lymphoblastic leukemia/lymphoma	hsa-mir-29b-1
Precursor cell lymphoblastic leukemia/lymphoma	hsa-mir-29b-2
Precursor cell lymphoblastic leukemia/lymphoma	hsa-mir-29c
Precursor cell lymphoblastic leukemia/lymphoma	hsa-mir-34a
Precursor cell lymphoblastic leukemia/lymphoma	hsa-mir-708
Precursor cell lymphoblastic leukemia/lymphoma	hsa-mir-99a
Precursor T-cell lymphoblastic leukemia/lymphoma	hsa-mir-223

Table S2

miRNA	Target Gene	Target Gene (Entrez ID)	References (PMID)	Role	Phenotype association	Phenotype group
hsa-miR-125b-5p	TP53	7157	20216554			
hsa-miR-125b-5p	TP53	7157	19293287	Apoptosis in brain		
hsa-miR-125b-5p	TP53	7157	21935352	Apoptosis and proliferation		
hsa-miR-125b-5p	TP53	7157	23497288	Chemoresistance	Ewing sarcoma	Cancer
hsa-miR-125b-5p	TP53	7157	22331826	Mesenchymal protection from anoikis		
hsa-miR-125b-5p	TP53	7157	26585673	Myofibroblast transition	Cardiac fibrosis	Other
hsa-miR-125b-5p	CBFB	865	17891175	Downregulated in prostate cancer	Prostate cancer	Cancer
hsa-miR-125b-5p	CBFB	865	21903586		Prostate cancer	Cancer
hsa-miR-125b-5p	CBFB	865	22689670	Myeloid cell line transformation	Leukemia	Leukemia
hsa-miR-125b-5p	IRF4	3662	20497960	B cell differentiation inhibition		
hsa-miR-125b-5p	IRF4	3662	23446348	Gene expression regulation		
hsa-miR-125b-5p	IRF4	3662	21572407			
hsa-miR-125b-5p	IRF4	3662	20371350			
hsa-miR-125b-5p	IRF4	3662	27903272	Gene expression regulation	Multiple myeloma	Cancer
hsa-miR-125b-5p	CDKN2A	1029	20347935	Astrogliosis/glia cell proliferation	Astrogliosis	Other
hsa-miR-125b-5p	CDKN2A	1029	23585871	Gene expression regulation		
hsa-miR-125b-5p	BCL2	596	22293115	Apoptosis induction	Hepatocellular carcinoma	Cancer
hsa-miR-125b-5p	BCL2	596	26057745	Mitochondrial apoptosis		
hsa-miR-125b-5p	NPM1	4869	23622248	Gene expression regulation		
hsa-miR-125b-5p	CEBPA	1050	23622248	Gene expression regulation		
hsa-miR-125b-5p	CEBPA	1050	25982911	Gene expression regulation	Acute myeloid leukemia	Leukemia
hsa-miR-125b-5p	CREBBP	1387	23622248	Gene expression regulation		
hsa-miR-125b-5p	ABL1	25	23622248	Gene expression regulation		
hsa-miR-125b-5p	TET2	54790	24120864	Hematopoiesis	Malignant hematopoiesis	Hematopoiesis
hsa-miR-125b-2-3p	NSD1	64324	23313552	Gene expression regulation		
hsa-miR-125b-1-3p	TP53	7157	27592685	Genotoxic stress response	Breast carcinoma	Cancer
hsa-miR-125b-5p	JAK2	3717	26011312	Polycythemia vera, thrombocythemia	Polycythemia vera, thrombocythemia	Other

hsa-miR-142-3p	KMT2A	4297	22473208	Gene expression regulation	Epstein-Barr virus	Other
hsa-miR-142-3p	KMT2A	4297	22291592	Gene expression regulation	Epstein-Barr virus	Other
hsa-miR-142-3p	GFI1	2672	22473208	Gene expression regulation	Epstein-Barr virus	Other
hsa-miR-155-5p	CBFB	865	18668040	Gene expression regulation		
hsa-miR-155-5p	SPI1	6688	19386588	Pathogen binding modulation		
hsa-miR-155-5p	SPI1	6688	20680360	Gene expression regulation	Marek's disease	Other
hsa-miR-155-5p	SPI1	6688	18668040	Gene expression regulation		
hsa-miR-155-5p	SPI1	6688	25288398	Terminal B cell differentiation		
hsa-miR-155-5p	SPI1	6688	22473208	Gene expression regulation	Epstein-Barr virus	Other
hsa-miR-155-5p	SPI1	6688	25092144	Gene expression regulation	Acute myeloid leukemia	Leukemia
hsa-miR-155-5p	KRAS	3845	19193853	Interleukin signaling pathway modulation		
hsa-miR-155-5p	KRAS	3845	18668040	Gene expression regulation		
hsa-miR-155-5p	KRAS	3845	20584899	Gene expression regulation		
hsa-miR-155-5p	KRAS	3845	22473208	Gene expression regulation	Epstein-Barr virus	Other
hsa-miR-155-5p	MYB	4602	21062812	Gene expression regulation	B-cell lymphoma	Cancer
hsa-miR-155-5p	MYB	4602	20680360	Gene expression regulation	Marek's disease	Other
hsa-miR-155-5p	MYB	4602	23807165	Paclitaxel resistance	Ovarian cancer	Cancer
hsa-miR-155-5p	PRKAR1A	5573	20584899	Gene expression regulation		
hsa-miR-155-5p	PHF6	84295	18668040	Gene expression regulation		
hsa-miR-155-5p	CDKN2A	1029	18668040	Gene expression regulation		
hsa-miR-155-5p	IRF2BP2	359948	22473208	Gene expression regulation	Epstein-Barr virus	Other
hsa-miR-155-5p	CBL	867	22473208	Gene expression regulation	Epstein-Barr virus	Other
hsa-miR-155-3p	WT1	7490	23824327			
hsa-miR-155-3p	FBXW7	55294	27306418	Cancer promotion	Hepatocellular carcinoma	Cancer
hsa-miR-155-3p	TP53	7157	27903673	Chemoresistance	Lung cancer	Cancer
hsa-miR-155-5p	FBXW7	55294	27904771	Role in tumor formation	Glioma	Cancer
hsa-miR-15a-5p	MYB	4602	18818396	Autoregulatory feedback loop	Myeloid leukemia	Leukemia
hsa-miR-15a-5p	MYB	4602	21205891	Elevate fetal hemoglobin expression	Trisomy 13	Hematopoiesis
hsa-miR-15a-5p	MYB	4602	28337280	Tumor suppression, cell migration	Hepatocarcinoma	Cancer
hsa-miR-15a-5p	BCL2	596	18362358	Gene expression regulation	Chronic lymphocytic leukemia	Leukemia

hsa-miR-15a-5p	BCL2	596	17707831	Differential expression	Chronic lymphocytic leukemia	Leukemia
hsa-miR-15a-5p	BCL2	596	19478946	Gene expression regulation		
hsa-miR-15a-5p	BCL2	596	20876285	Chemoresistance	Breast tumor	Cancer
hsa-miR-15a-5p	BCL2	596	17260024	Gene expression regulation	Acute promyelocytic leukemia	Leukemia
hsa-miR-15a-5p	BCL2	596	16166262	Apoptosis induction	Chronic lymphocytic leukemia	Leukemia
hsa-miR-15a-5p	BCL2	596	19903841	Gene expression regulation	Ovarian cancer	Cancer
hsa-miR-15a-5p	BCL2	596	22473208	Gene expression regulation	Epstein-Barr virus	Other
hsa-miR-15a-5p	BCL2	596	26397135	Chemoresistance	Breast cancer	Cancer
hsa-miR-15a-5p	WT1	7490	18362358	Differential expression	Chronic lymphocytic leukemia	Leukemia
hsa-miR-15a-5p	TP53	7157	21205967	Feedback signaling	B-cell chronic lymphocytic leukemia	Leukemia
hsa-miR-15a-5p	IRF4	3662	22473208	Gene expression regulation	Epstein-Barr virus	Other
hsa-miR-15a-5p	ZBTB16	7704	20371350			
hsa-miR-15a-5p	ASXL1	171023	22473208	Gene expression regulation	Epstein-Barr virus	Other
hsa-miR-15a-3p	BCL2	596	27596960	Cell growth, angiogenesis, anti-tumor activity	Multiple myeloma	Cancer
hsa-miR-16-1-3p	BCL2	596	27596960	Cell growth, angiogenesis, anti-tumor activity	Multiple myeloma	Cancer
hsa-miR-16-1-3p	NPM1	4869	26701625			
hsa-miR-17-5p	BCL2	596	19666108	Neuronal differentiation		
hsa-miR-17-5p	BCL2	596	24280866	Differential expression	Acute lymphoblastic leukemia	Leukemia
hsa-miR-17-5p	RUNX1	861	17589498	Monocytopoiesis	Hematopoiesis	Hematopoiesis
hsa-miR-17-5p	CBL	867	23622248	Gene expression regulation		
hsa-miR-17-5p	FANCA	2175	23622248	Gene expression regulation		
hsa-miR-17-5p	KMT2A	4297	23622248	Gene expression regulation		
hsa-miR-17-5p	PHF6	84295	22473208	Gene expression regulation	Epstein-Barr virus	Other
hsa-miR-17-5p	TP53	7157	24955218	Differential expression	Osteosarcoma	Cancer
hsa-miR-17-5p	PRKAR1A	5573	22012620	Differential expression		
hsa-miR-17-3p	ABL1	25	26701625			
hsa-miR-181b-5p	BCL2	596	20162574	Chemoresistance	Gastric cancer, lung cancer	Cancer
hsa-miR-181b-5p	BCL2	596	22610076	Drug sensitivity	Chronic lymphocytic leukemia	Leukemia
hsa-miR-181b-5p	BCL2	596	23440261	Drug sensitivity	Pancreatic ductal adenocarcinoma	Cancer

hsa-miR-181a-5p	BCL2	596	20162574			
hsa-miR-181a-5p	BCL2	596	22285729	Drug sensitivity	Multidrug-resistant leukemia	Leukemia
hsa-miR-181a-5p	BCL2	596	22209977	Drug sensitivity	Acute myeloid leukemia	Leukemia
hsa-miR-181a-5p	BCL2	596	21958558	Influences apoptosis		
hsa-miR-181a-5p	BCL2	596	22610076			
hsa-miR-181a-5p	BCL2	596	24335172	Drug sensitivity	Breast cancer	Cancer
hsa-miR-181a-5p	BCL2	596	24002437	Astrocyte death	Ischemia	Other
hsa-miR-181a-5p	BCL2	596	27802900	Apoptosis regulation	Acute respiratory distress syndrome	Other
hsa-miR-181a-5p	ATM	472	21274007	Differential expression	Head and neck squamous cell carcinoma	Cancer
hsa-miR-181a-5p	ATM	472	23656790	DNA damage response	Breast cancer	Cancer
hsa-miR-181a-5p	ATM	472	24531888	Oncogenous	Gastric cancer	Cancer
hsa-miR-181a-5p	ATM	472	22473208	Gene expression regulation	Epstein-Barr virus	Other
hsa-miR-181a-5p	ATM	472	20371350			
hsa-miR-181a-5p	ATM	472	26113450	Cell transition and proliferation	Acute myeloid leukemia	Leukemia
hsa-miR-181a-5p	HRAS	3265	21167132	Tumor suppressive effects	Squamous cell carcinoma	Cancer
hsa-miR-181a-5p	PTPN11	5781	17382377	T cell sensitivity		
hsa-miR-181a-5p	KRAS	3845	20371350			
hsa-miR-181a-5p	KRAS	3845	23752186	Pathway promotion	Pancreatic ductal adenocarcinoma	Cancer
hsa-miR-181a-5p	KRAS	3845	27517749	Pathway promotion	Acute myeloid leukemia	Leukemia
hsa-miR-181a-5p	NOTCH1	4851	22916024	Oncogenous	Acute lymphoblastic leukemia	Leukemia
hsa-miR-181b-5p	ATM	472	23656790	DNA damage response	Breast cancer	Cancer
hsa-miR-181b-5p	ATM	472	22473208	Gene expression regulation	Epstein-Barr virus	Other
hsa-miR-181b-5p	ATM	472	20371350			
hsa-miR-181a-5p	TERT	7015	25444904	Multiple anti-cancer effects	T-cell leukemia	Leukemia
hsa-miR-181a-2-3p	WT1	7490	22012620	Bone development		
hsa-miR-181b-3p	PLCG2	5336	23824327			
hsa-miR-181b-2-3p	PLCG2	5336	23824327			
hsa-miR-181a-5p	NRAS	4893	27517749	Pathway promotion	Acute myeloid leukemia	Leukemia
hsa-miR-181a-5p	RUNX1	861	26580398	Feedback signaling	Acute lymphoblastic leukemia	Leukemia
hsa-miR-181a-5p	CEBPA	1050	27673564	Macrophage phenotype modulation	Multiple cancers	Cancer

hsa-miR-18a-3p	KRAS	3845	19372139	Tumor suppressor	Carcinoma	Cancer
hsa-miR-18a-5p	ATM	472	21980462	DNA damage response	Breast cancer	Cancer
hsa-miR-18a-5p	ATM	472	23437304	DNA damage response	Colorectal cancer	Cancer
hsa-miR-18a-5p	ATM	472	20371350			
hsa-miR-18a-5p	ATM	472	23857602	Gene expression regulation	Breast cancer	Cancer
hsa-miR-18a-5p	ATM	472	22100165	miRNA targeting	Lymphoma	Cancer
hsa-miR-18a-5p	ATM	472	22291592			
hsa-miR-18a-5p	ATM	472	23818585	Pathway promotion	Sarcoma	Cancer
hsa-miR-18a-3p	DNMT3A	1788	23622248			
hsa-miR-18a-5p	BCL2	596	24280866	Differential expression	Acute lymphoblastic leukemia	Leukemia
hsa-miR-18a-5p	TP53	7157	24955218	Differential expression	Osteosarcoma	Cancer
hsa-miR-18a-5p	RUNX1	861	25452107	Blood-tumor barrier permeability	Glioma	Cancer
hsa-miR-18a-3p	ATM	472	25963391	Radiosensitivity	Cervical cancer	Cancer
hsa-miR-196b-5p	BCL2	596	23293219	Apoptosis, proliferation	endometriotic stromal cells	Other
hsa-miR-196b-5p	GFI1	2672	24334453	Gene expression regulation	Acute myeloid leukemia	Leukemia
hsa-miR-19a-3p	KIT	3815	21880628	Gene expression regulation	Acute myeloid leukemia	Leukemia
hsa-miR-19a-3p	MLLT10	8028	23592263	Gene targeting	HIV	Other
hsa-miR-19a-3p	MLLT10	8028	27292025	Gene targeting	Prostate cancer	Cancer
hsa-miR-19a-3p	NF1	4763	22473208	Gene expression regulation	Epstein-Barr virus	Other
hsa-miR-19a-3p	IKZF1	10320	22473208	Gene expression regulation	Epstein-Barr virus	Other
hsa-miR-19a-3p	STAT5B	6777	23313552			
hsa-miR-19a-5p	FANCF	2188	23824327			
hsa-miR-19a-3p	TP53	7157	22473208	Gene expression regulation	Epstein-Barr virus	Other
hsa-miR-19b-2-5p	FANCF	2188	23824327			
hsa-miR-19b-1-5p	FANCF	2188	23824327			
hsa-miR-20a-5p	BCL2	596	19666108	Cell differentation		
hsa-miR-20a-5p	RUNX1	861	17589498	Monocytopoiesis		Hematopoiesis
hsa-miR-20a-5p	NRAS	4893	19110058	Gene expression regulation	Alzheimer's disease	Other
hsa-miR-20a-5p	KIT	3815	21880628	Gene expression regulation	Acute myeloid leukemia	Leukemia
hsa-miR-20a-3p	NF1	4763	23622248			
hsa-miR-20a-5p	NUP214	8021	23622248			
hsa-miR-20a-5p	PHF6	84295	22473208	Gene expression regulation	Epstein-Barr virus	Other

hsa-miR-20a-5p	TP53	7157	24955218	Differential expression	Osteosarcoma	Cancer
hsa-miR-20a-5p	PRKAR1A	5573	22012620	Differential expression		
hsa-miR-221-3p	KIT	3815	19088079	Growth factor signaling		
hsa-miR-221-3p	KIT	3815	19126397	Gene expression regulation	Cutaneous melanoma	Cancer
hsa-miR-221-3p	KIT	3815	18246122	TRAIL resistance	Non-small cell lung cancer	Cancer
hsa-miR-221-3p	KIT	3815	18983236	Gene expression regulation	Melanoma	Cancer
hsa-miR-221-3p	KIT	3815	18417445	Pathway promotion	Melanoma	Cancer
hsa-miR-221-3p	KIT	3815	16365291	Gene expression regulation	Papillary thyroid carcinoma	Cancer
hsa-miR-221-3p	KIT	3815	20018759	Gene expression regulation	Hepatocellular carcinoma	Cancer
hsa-miR-221-3p	KIT	3815	16330772	Erythropoiesis inhibition	Erythropoiesis	Hematopoiesis
hsa-miR-221-3p	TP53	7157	21226887	Gene expression regulation	Oral carcinoma cells	Cancer
hsa-miR-221-3p	PALB2	79728	23622248			
hsa-miR-221-3p	RUNX1	861	24329418		Chronic ethanol consumption	Other
hsa-miR-221-5p	ABL1	25	26701625			
hsa-miR-222-3p	KIT	3815	18417445			
hsa-miR-222-3p	KIT	3815	18246122	TRAIL resistance	Non-small cell lung cancer	Cancer
hsa-miR-222-3p	KIT	3815	18983236	Gene expression regulation	Melanoma	Cancer
hsa-miR-222-3p	KIT	3815	16365291	Gene expression regulation	Papillary thyroid carcinoma	Cancer
hsa-miR-222-3p	KIT	3815	16330772	Erythropoiesis inhibition	Erythropoiesis	Hematopoiesis
hsa-miR-222-3p	KIT	3815	23969726	Gene expression regulation	Gastrointestinal stromal tumors	Cancer
hsa-miR-222-3p	TP53	7157	21226887	Gene expression regulation	Oral carcinoma cells	Cancer
hsa-miR-222-3p	TP53	7157	23622248			
hsa-miR-222-3p	IRF2BP2	359948	23622248			
hsa-miR-223-3p	FBXW7	55294	22270966	Oncogenous	Gastric cancer	Cancer
hsa-miR-223-3p	FBXW7	55294	24324762	Gene expression regulation		
hsa-miR-223-3p	FBXW7	55294	25888377	Chemoresistance	Gastric cancer	Cancer
hsa-miR-223-3p	FBXW7	55294	25159729	Drug sensitivity	Gastric cancer	Cancer
hsa-miR-223-3p	FBXW7	55294	28000896	Oncogenous	Testicular germ cell tumors	Cancer
hsa-miR-223-3p	TAL1	6886	23857984	Gene expression regulation	Acute lymphoblastic leukemia	Leukemia
hsa-miR-223-3p	ATM	472	24606854	Radiosensitivity	Unspecified tumors	Cancer
hsa-miR-223-3p	TP53	7157	23592263		HIV	Other
hsa-miR-223-5p	ETV6	2120	23446348	Gene expression regulation		

hsa-miR-223-5p	POT1	25913	21572407			
hsa-miR-223-5p	FBXW7	55294	27398136	Drug sensitivity	Non-small cell lung cancer	Cancer
hsa-miR-29a-3p	DNMT3A	1788	17890317	Methylation	Lung cancer	Cancer
hsa-miR-29a-3p	DNMT3A	1788	20643754	Apoptosis		
hsa-miR-29a-3p	DNMT3A	1788	26251039	Differential expression	T-cell acute lymphoblastic leukemia	Leukemia
hsa-miR-29a-3p	BCL2	596	20041405	Apoptosis	Hepatocellular carcinoma	Cancer
hsa-miR-29a-3p	BCL2	596	25006537	Differential expression	Acute myeloid leukemia	Leukemia
hsa-miR-29a-3p	ABL1	25	23428668	Apoptosis	Chronic myeloid leukemia	Leukemia
hsa-miR-29a-3p	TET2	54790	23820384	Gene expression regulation		
hsa-miR-29a-3p	TET2	54790	26251039	Differential expression	T-cell acute lymphoblastic leukemia	Leukemia
hsa-miR-29a-3p	TET2	54790	26404510	Gene expression regulation	Prostate cancer	Cancer
hsa-miR-29b-2-5p	IRF2BP2	359948	23446348			
hsa-miR-34a-5p	MYB	4602	19584398	Cell differentation		
hsa-miR-34a-5p	MYB	4602	21566225			
hsa-miR-34a-5p	MYB	4602	24504520		Leukemia	Leukemia
hsa-miR-34a-5p	BCL2	596	18803879	Tumor suppression	Gastric cancer	Cancer
hsa-miR-34a-5p	BCL2	596	19683563	Differential expression	Alzheimer's disease	Other
hsa-miR-34a-5p	BCL2	596	18505919	Tumor suppression	Neuroblastoma	Cancer
hsa-miR-34a-5p	BCL2	596	19461653			
hsa-miR-34a-5p	BCL2	596	17914404	Tumor suppression		
hsa-miR-34a-5p	BCL2	596	17656095	Tumor suppression	Non-small cell lung cancer	Cancer
hsa-miR-34a-5p	BCL2	596	20598588	Cell differentation	B lyhmphocyte development	Hematopoiesis
hsa-miR-34a-5p	BCL2	596	21399894	Chemoresistance	Breast cancer	Cancer
hsa-miR-34a-5p	BCL2	596	21566225	Differential expression		
hsa-miR-34a-5p	BCL2	596	24565525	Differential expression		
hsa-miR-34a-5p	BCL2	596	23862748	Drug sensitivity	Hepatocellular carcinoma	Cancer
hsa-miR-34a-5p	BCL2	596	23292172	Antitumor activity	Tumor model	Cancer
hsa-miR-34a-5p	BCL2	596	26802970	Gene expression regulation	Hearing loss	Other
hsa-miR-34a-5p	BCL2	596	27939626	Apoptosis		
hsa-miR-34a-5p	BCL2	596	26406332		Non-small cell lung cancer	Cancer
hsa-miR-34a-5p	BCL2	596	25910896	Apoptosis		
hsa-miR-34a-5p	NOTCH1	4851	14697198			

hsa-miR-34a-5p	NOTCH1	4851	19461653			
hsa-miR-34a-5p	NOTCH1	4851	20351093	Invasion suppression	Choriocarcinoma, cervical carcinoma	Cancer
hsa-miR-34a-5p	NOTCH1	4851	19773441	Gene expression regulation	Glioblastoma, medulloblastoma, glioma	Cancer
hsa-miR-34a-5p	NOTCH1	4851	17150773	Cell differentation		
hsa-miR-34a-5p	NOTCH1	4851	22363487	Pathway promotion		
hsa-miR-34a-5p	NOTCH1	4851	22684561	Cell migration, invasion	Urothelial bladder carcinoma	Cancer
hsa-miR-34a-5p	NOTCH1	4851	23085450	Drug sensitivity	Breast cancer	Cancer
hsa-miR-34a-5p	NOTCH1	4851	23226240	Cell differentation		
hsa-miR-34a-5p	NOTCH1	4851	22438124	Pathway suppression	Non-small cell lung cancer	Cancer
hsa-miR-34a-5p	NOTCH1	4851	23035210	Mimics a therapeutic agent	Multiple myeloma	Cancer
hsa-miR-34a-5p	NOTCH1	4851	23430952	Pathway suppression	Osteosarcoma	Cancer
hsa-miR-34a-5p	NOTCH1	4851	28145431	Cell invasion	Pancreatic cancer	Cancer
hsa-miR-34a-5p	NOTCH1	4851	26493107	Gene expression regulation	Neointima	Other
hsa-miR-34a-5p	NOTCH1	4851	28129650	Oncogenous	Liver cancer	Cancer
hsa-miR-34a-5p	SPI1	6688	20598588			
hsa-miR-34a-5p	TP53	7157	23292869	Differential expression	Breast cancer	Cancer
hsa-miR-34a-5p	TP53	7157	26406332		Non-small cell lung cancer	Cancer
hsa-miR-34a-5p	TP53	7157	26403328	Differential expression	Alcoholic hepatitis	Other
hsa-miR-34a-5p	TP53	7157	26177460	Gene expression regulation		
hsa-miR-34a-5p	RRAS	6237	20371350			
hsa-miR-34a-5p	LEF1	51176	21566225	Gene expression regulation		
hsa-miR-34a-5p	LEF1	51176	25587085	Gene expression regulation	Prostate cancer	Cancer
hsa-miR-34a-5p	LEF1	51176	28098757	Cell proliferation	Primary myelofibrosis	Hematopoiesis
hsa-miR-34a-5p	CDKN2A	1029	21128241	Differential expression	HPV	Other
hsa-miR-34a-3p	BCL2	596	28340489	Cell proliferation and apoptosis	Meningioma	Cancer
hsa-miR-34a-5p	KIT	3815	24009080	Drug sensitivity		
hsa-miR-34a-5p	KIT	3815	27056900	Chemoresistance	Osteosarcoma	Cancer
hsa-miR-663a	HRAS	3265	23953123	Gene expression regulation		
hsa-miR-663a	TP53	7157	27105517	Apoptosis	Breast ductal carcinoma	Cancer
hsa-miR-663a	ABL1	25	26701625			
hsa-miR-92a-2-5p	TP53	7157	24955218	Differential expression	Osteosarcoma	Cancer

hsa-miR-92a-1-5p	TP53	7157	24955218	Differential expression	Osteosarcoma	Cancer
hsa-miR-92a-2-5p	SETBP1	26040	23446348			
hsa-miR-92a-1-5p	FBXW7	55294	26701625			
hsa-miR-92a-1-5p	FBXW7	55294	27292025			
hsa-miR-92a-2-5p	KMT2A	4297	26701625			

Table S3

miRNA	Target Gene	Target Gene (Entrez ID)	References (PMID)	Role	Phenotype association	Phenotype group	Additional information
hsa-miR-125b-5p	CBFB	865	22689670	Myeloid cell line transformation	Leukemia	Leukemia	Based on animal models - can induce leukemia
hsa-miR-125b-5p	CEBPA	1050	25982911	Gene expression regulation	Acute myeloid leukemia	Leukemia	\
hsa-miR-155-5p	SPI1	6688	25092144	Gene expression regulation	Acute myeloid leukemia	Leukemia	Cell lines
hsa-miR-15a-5p	MYB	4602	18818396	Autoregulatory feedback loop	Myeloid leukemia	Leukemia	Cell lines
hsa-miR-15a-5p	BCL2	596	18362358	Gene expression regulation	Chronic lymphocytic leukemia	Leukemia	Cell lines
hsa-miR-15a-5p	BCL2	596	17707831	Differential expression	Chronic lymphocytic leukemia	Leukemia	\
hsa-miR-15a-5p	BCL2	596	17260024	Gene expression regulation	Acute promyelocytic leukemia	Leukemia	Patient study
hsa-miR-15a-5p	BCL2	596	16166262	Apoptosis induction	Chronic lymphocytic leukemia	Leukemia	Patient study
hsa-miR-15a-5p	WT1	7490	18362358	Differential expression	Chronic lymphocytic leukemia	Leukemia	Cell lines, tissue samples
hsa-miR-15a-5p	TP53	7157	21205967	Feedback signaling	B-cell chronic lymphocytic leukemia	Leukemia	Cell lines, tissue samples
hsa-miR-17-5p	BCL2	596	24280866	Differential expression	Acute lymphoblastic leukemia	Leukemia	Patient study
hsa-miR-181b-5p	BCL2	596	22610076	Drug sensitivity	Chronic lymphocytic leukemia	Leukemia	Patient study
hsa-miR-181a-5p	BCL2	596	22285729	Drug sensitivity	Multidrug-resistant leukemia	Leukemia	Cell lines
hsa-miR-181a-5p	BCL2	596	22209977	Drug sensitivity	Acute myeloid leukemia	Leukemia	Cell lines
hsa-miR-181a-5p	ATM	472	26113450	Cell transition and proliferation	Acute myeloid leukemia	Leukemia	Patient study
hsa-miR-181a-5p	KRAS	3845	27517749	Pathway promotion	Acute myeloid leukemia	Leukemia	\
hsa-miR-181a-5p	NOTCH1	4851	22916024	Oncogenous	Acute lymphoblastic leukemia	Leukemia	Cell lines, animal models
hsa-miR-181a-5p	TERT	7015	25444904	Multiple anti-cancer effects	T-cell leukemia	Leukemia	Cell lines
hsa-miR-181a-5p	NRAS	4893	27517749	Pathway promotion	Acute myeloid leukemia	Leukemia	Cell lines
hsa-miR-181a-5p	RUNX1	861	26580398	Feedback signaling	Acute lymphoblastic leukemia	Leukemia	Animal models
hsa-miR-18a-5p	BCL2	596	24280866	Differential expression	Acute lymphoblastic leukemia	Leukemia	Cell lines
hsa-miR-196b-5p	GFI1	2672	24334453	Gene expression regulation	Acute myeloid leukemia	Leukemia	Animal models
hsa-miR-19a-3p	KIT	3815	21880628	Gene expression regulation	Acute myeloid leukemia	Leukemia	Tissue samples
hsa-miR-20a-5p	KIT	3815	21880628	Gene expression regulation	Acute myeloid leukemia	Leukemia	Tissue samples
hsa-miR-223-3p	TAL1	6886	23857984	Gene expression regulation	Acute lymphoblastic leukemia	Leukemia	\
hsa-miR-29a-3p	DNMT3A	1788	26251039	Differential expression	T-cell acute lymphoblastic leukemia	Leukemia	\

hsa-miR-29a-3p	BCL2	596	25006537	Differential expression	Acute myeloid leukemia	Leukemia	\
hsa-miR-29a-3p	ABL1	25	23428668	Apoptosis	Chronic myeloid leukemia	Leukemia	\
hsa-miR-29a-3p	TET2	54790	26251039	Differential expression	T-cell acute lymphoblastic leukemia	Leukemia	\
hsa-miR-34a-5p	MYB	4602	24504520	\	Leukemia	Leukemia	Computational study
hsa-miR-100-5p	CTDSPL	10217	21643017	Cell differentiation	Acute myeloid leukemia	Leukemia	Cell lines
hsa-miR-199b-5p	PODXL	5420	22374871	Gene expression regulation, differential expression	Acute myeloid leukemia	Leukemia	Cell lines
hsa-miR-199b-5p	DDR1	780	22374871	Gene expression regulation, differential expression	Acute myeloid leukemia	Leukemia	Cell lines
hsa-miR-26a-5p	E2F7	144455	23096114	Cell proliferation	Acute myeloid leukemia	Leukemia	Cell lines
hsa-miR-34b-3p	CREB1	1385	19258499	Gene expression regulation	Acute myeloid leukemia	Leukemia	Cell lines
hsa-miR-17-5p	TCEAL1	9338	23059786	Gene expression regulation	Acute myeloid leukemia	Leukemia	Cell lines
hsa-miR-20a-5p	TCEAL1	9338	23059786	Gene expression regulation	Acute myeloid leukemia	Leukemia	Cell lines
hsa-miR-15a-5p	BCL2	596	16166262	Apoptosis	Chronic lymphocytic leukemia	Leukemia	Cell lines
hsa-miR-16-5p	BCL2	596	16166262	Apoptosis	Chronic lymphocytic leukemia	Leukemia	Cell lines
hsa-miR-15a-5p	TP53	7157	21205967	Pathogenic pathways	Chronic lymphocytic leukemia	Leukemia	Cell lines
hsa-miR-16-5p	TP53	7157	21205967	Pathogenic pathways	Chronic lymphocytic leukemia	Leukemia	Cell lines
hsa-miR-34a-3p	MYB	4602	24504520	Gene expression regulation	Acute myeloid leukemia	Leukemia	Patient study
hsa-miR-21-5p	PTEN	5728	25361012	Gene expression regulation	Chronic lymphocytic leukemia	Leukemia	Patient study
hsa-miR-181a-5p	BCL2	596	22285729	Drug sensitivity	Multidrug-resistant leukemia	Leukemia	Cell lines
hsa-miR-181a-5p	BCL2	596	22610076	Drug sensitivity	Chronic lymphocytic leukemia	Leukemia	Cell lines
hsa-miR-219a-5p	TRIB3	57761	24974767	Treatment sensitivity	T-cell acute leukemia	Leukemia	Cell lines
hsa-miR-654-3p	EXOG	9941	24974767	Treatment sensitivity	T-cell acute leukemia	Leukemia	Cell lines
hsa-miR-219a-5p	MTIF	4494	24974767	Treatment sensitivity	T-cell acute leukemia	Leukemia	Cell lines
hsa-miR-27b-3p	CCNA2	890	24974217	Cell proliferation	Acute leukemia	Leukemia	Animal model
hsa-miR-125b-5p	PCTP	58488	22723551	Cell metabolism	Chronic lyhmphocytic leukemia	Leukemia	\
hsa-miR-125b-5p	LIPA	3988	22723551	Cell metabolism	Chronic lyhmphocytic leukemia	Leukemia	\
hsa-miR-125b-5p	GSS	2937	22723551	Cell metabolism	Chronic lyhmphocytic leukemia	Leukemia	\
hsa-miR-125b-5p	IKZF2	22807	22723551	Cell metabolism	Chronic lyhmphocytic leukemia	Leukemia	\
hsa-miR-125b-5p	IKZF3	22806	22723551	Cell metabolism	Chronic lyhmphocytic leukemia	Leukemia	\
hsa-miR-125b-5p	IKZF4	64375	22723551	Cell metabolism	Chronic lyhmphocytic leukemia	Leukemia	\
hsa-miR-370-3p	FOXM1	2305	22900969	Tumor suppression	Acute myeloid leukemia	Leukemia	\

hsa-miR-155	HDAC4	9759	23169640	Gene expression regulation	Nonspecific leukemia	Leukemia	Animal model
hsa-miR-155	BCL6	604	23169640	Gene expression regulation	Nonspecific leukemia	Leukemia	Animal model
hsa-miR-27a-3p	YWHAZ	7534	23236401	Tumor suppression	Acute leukemia	Leukemia	\
hsa-miR-196b-5p	HOXA9	3205	23894305	Gene expression regulation	Chronic myeloid leukemia	Leukemia	\
hsa-miR-29a-3p	CCND2	894	24076586	Tumor suppression	Acute myeloid leukemia	Leukemia	\
hsa-miR-29a-3p	AKT2	208	24076586	Tumor suppression	Acute myeloid leukemia	Leukemia	\
hsa-miR-150-5p	MYB	4602	24086639	Cell differentiation	Acute myeloid leukemia	Leukemia	\
hsa-miR-370-3p	FOXM1	2305	24148180	Drug sensitivity	Acute myeloid leukemia	Leukemia	\

Figure S1

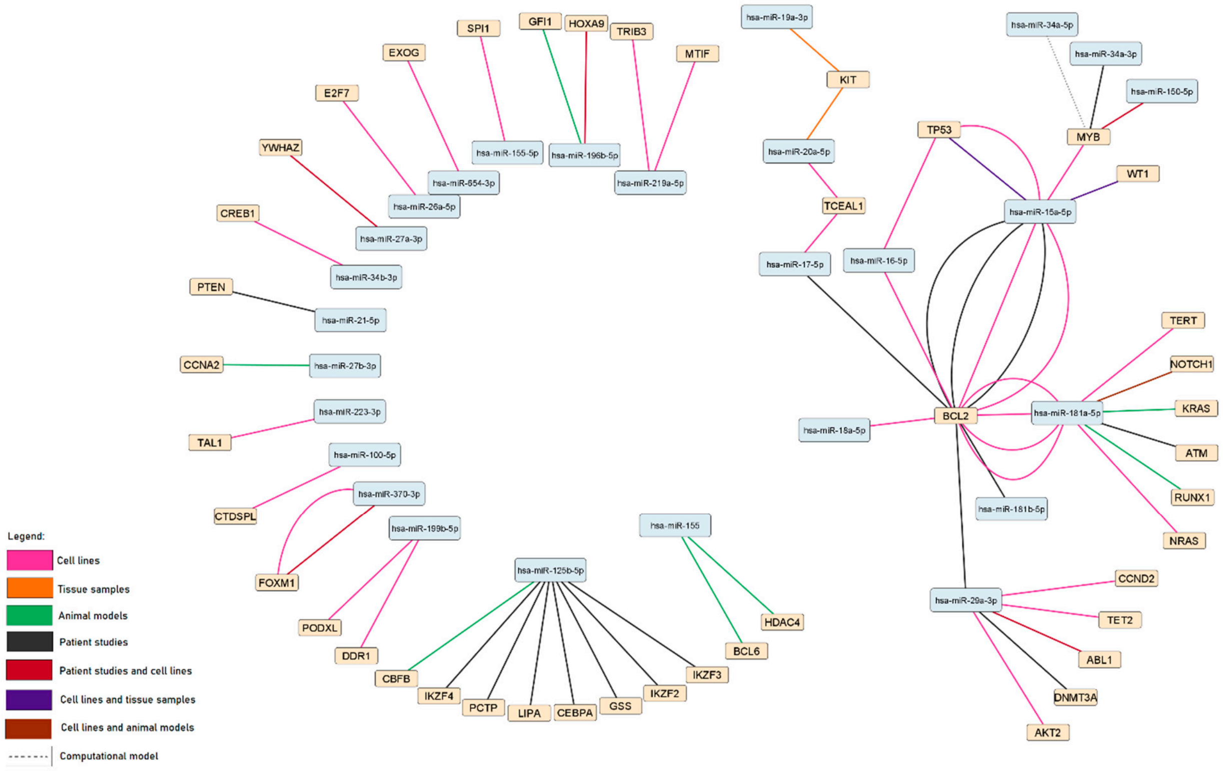

Supplement: Supplementary file 1 [file ijms-23-03469-s001.zip › ijms-1625503-supplementary.pdf]
